# Iran Diabetes Research Roadmap (IDRR): the study protocol

**DOI:** 10.1186/s40200-016-0277-z

**Published:** 2016-12-15

**Authors:** Gita Shafiee, Ensieh Nasli-Esfahani, Fatemeh Bandarian, Maryam Peimani, Bahareh Yazdizadeh, Farideh Razi, Farshad Farzadfar, Bagher Larijani

**Affiliations:** 1 Chronic Diseases Research Center, Endocrinology and Metabolism Population Sciences Institute, Tehran University of Medical Sciences, Tehran, Iran; 2 Diabetes Researcher Center, Endocrinology and Metabolism Clinical Sciences Institute, Tehran University of Medical Sciences, 5th floor, Diabetes Clinic, Cross Heyat Ave., Shahrivar Ave., North Kargar St., Tehran, Iran; 3 Knowledge Utilization Research Center, Tehran University of Medical Sciences, Tehran, Iran; 4 Non-Communicable Diseases Research Center, Endocrinology and Metabolism Population Sciences Institute, Tehran University of Medical Sciences, Tehran, Iran; 5 Endocrinology and Metabolism Researcher Center, Endocrinology and Metabolism Clinical Sciences Institute, Tehran University of Medical Sciences, Tehran, Iran

**Keywords:** Roadmap, Diabetes mellitus, Protocol

## Abstract

**Background:**

Diabetes mellitus is a common metabolic disorder which is increasing worldwide. This study aimed to undertake a survey of the diabetes research in Iran to identify gaps and highlight strengths in order to develop a roadmap for diabetes research in Iran for the next years.

**Methods:**

To develop a roadmap and to identify major areas of diabetes research, we carried out a systematic assessment of Iranian diabetes research publications. All obtained studies were categorized to 11 groups and each group was classified according to the “study design”, “subject area”, “World Health Organization (WHO) classification”, and “Australian Standard Research Classifications”. The number of publications per each year was calculated. Research trends in publications in each area were assessed and compared.

**Conclusion:**

By this study, we will provide the highlighted priorities, the gaps of research and strategic mapping of each area of diabetes research that could serve as a precious guideline for national research initiatives in the field of diabetes. By a strategic map, we can achieve many advances in the all aspects of diabetes research that finally impact on the health, quality of life and well-being of diabetic patients.

## Background

Diabetes mellitus is one of the most important chronic diseases and the major health challenge worldwide. The prevalence of diabetes has reached epidemic proportions in most populations and it is expected to continue to rise [1]. The International Diabetes Foundation (IDF) estimates that 32 million adults in the Middle East and North Africa (MENA) currently have diabetes, accounting for 9.3% of the world’s adults with the disease and this number will double to 59.9 million by 2030 [2]. In a nationally representative’s report of diabetes in Iran, it is found that 11.37% of adults (2 million persons) have diabetes among whom 25% are not diagnosed [3]. Diabetes imposes a large economic burden on individuals and families, national health systems, and countries. The global health expenditures for prevention and care of diabetes, its complications, disability and premature mortality were USD 376 billion in 2010. By 2030, this number will exceed some USD 490 billion. The MENA region spends USD 5.5 billion annually on diabetes, accounting for 14% of its total health care expenditure [4]. In Iran, also, diabetes is a costly disease and consumes more than 8.6% of total health expenditure (USD 3.78 bilion) [5]. Diabetic patients have four times higher levels of hospitalization, 2.6 times higher numbers of annual physician visits and 2.5 times greater volume of drug prescriptions than non- diabetic patients [6].

These findings, along with the epidemiological and financial data, point to an imperative need to broaden research strategies for prevention and management of diabetes and its complications. Diabetes research have led to significant advances in understanding the causes and molecular pathways of diabetes and its complications, as well as new approaches and technologies for prevention and management. Remarkable progress has also been made on the development of sustainable health care delivery system. Despite significant advances in prevention and management of diabetes and its complications, there is currently no known cures or prevention for diabetes.

Therefore, a critical goal for research is to find ways that create lasting health benefits for people with or at risk for diabetes and alleviate the societal burden of this devastating disease. In this field, previous diabetes road maps charted in the United States and the Europe. In the United States, Australia, Canada and UK, diabetes road maps planed where the juvenile Diabetes Research Foundation (JDRE) has funded international research in type 1 diabetes and its complications since 1997 [7, 8].

In 2010, the Australian Type 1 Diabetes Research Agenda presented a strategic research plan designed to identify research strengths and define research priorities in the field. Another project “Type 1 Diabetes Research Roadmap” is also funded by JDRF to understand the strengths, weaknesses and opportunities in UK research [9]. A strategic mapping process for a large scale plan embracing all scientific areas has been used by the European Strategy Forum on Research Infrastructures (ESFRI). This map was organized in order to prepare future EU activities supporting the integration of and access to existing national research infrastructures [10].

The DIAMAP is one of the most important diabetes research maps in Europe. The DIAMAP provides strategic guidance for diabetes research activity in Europe for the benefit of all people with this disease [11].

Middle East is much more complicated geographically and politically than the Europe and USA. It is made up of individual nations, each with their own vastly differing research budgets, and many differences in research facilities. Health research challenges are also not necessarily the same for each country. Cosidering our country economic, social and cultural status and its differences with others, we need to have a strategic research map plan for own region to apply research findings in developing national guidelines [12].

Therefore, the Iranian Diabetes Research Network (IDRN) and its member universities of medical sciences across the country, decided to design a map for diabetes research. In June 2012, the Ministry of Health agreed to collaborate with IDRN to achieve a strategic plan for the sustainable development of diabetes research in Iran, with better coordination of research and more rational (science-based) use of funds, for the benefit of individuals with diabetes.

### Missions, goals and major objectives

The road map is intended to guide future investment in diabetes research by policy makers or researchers interested in this strategic plan. The map covers research areas considered by a group of leading experts to offer the greatest scientific interest and potential for advancing the field, ultimately for benefit to the patient. This new Iranian strategy for diabetes research is rational, science-driven and takes into consideration the unique expertise and opportunities in Iran.

The mission of roadmap is to chart the future of diabetes research in Iran for the benefit of all people with the disease. To this end, IDRN will undertake a survey of the diabetes research in Iran to identify gaps and highlight strengths in order to develop a strategic plan for diabetes research in Iran for the next years.

The specific objectives for the Iranian Diabetes Research Roadmap (IDRR) are:

- To create diabetes research databases from a survey of research activities in Iran.
- To compare this database with diabetes research activities in the world.
- To produce a strategic diabetes roadmap to guide future strategy for diabetes research in Iran.

## Methods

### Academic members and their activities

**Fig. 1:**
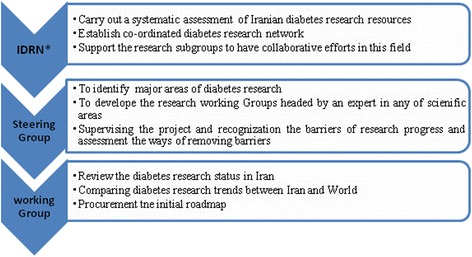
Flowchart of the “Iranian Diabetes Research Roadmap” members and their activities

Iranian Diabetes Research Roadmap has been coordinated by IDRN and overseen by a Project Steering Committee (PSC) of high-profile members of academic science which provides scientific guidance to the project (Fig. 1).

To achieve the main objectives of this project, we had an overview on diabetes research trends in the world and compared them with their counterparts in Iran. We have described the methods of this study in detail in our preliminary study [13].

The first step to draw a diabetes roadmap, was to undertake a wide survey on Iranian diabetes research and compare it with the world to identify gaps and highlight strength. Therefore, diabetes research has been considered in its broadest sense embracing, where possible, all aspects of this complex field, from molecules, cells and tissues, to whole animal studies, and clinical science and care. The steering committee suggested a list of 11 scientific areas of important opportunity related to diabetes. These areas are overlapping but complementary in scope:

- Diabetes management
- Patient education
- Physical activity
- Nutrition
- Diabetes prevention
- Diabetes Complications
- Diabetes comorbidity
- Gestational diabetes mellitus (GDM)
- Psychiatry
- Genetics
- Basic sciences

Based on the number of scientific areas, the diverse and talented group of researchers were voluntarily put into 11 groups. Each working group was chaired by an expert with comprehensive scientific knowledge in the relevant focus area and more broadly in diabetes research. Groups begin working when training workshops were held to explain the process and ensure that all members of each group receive the same information. Moreover, all researchers participated in the coordination workshop to share their experience. In these workshops they identified overarching goals to provide a broad vision considered important for advancement in the field. They additionally sent the project promotion report to the steering group monthly.

### Search strategy

This study included all studies published from the beginning until the first of 2015 in national and international journals by Iranian authors conducted in the field of diabetes. International databases including PubMed, Web of Science and SCOPUS as well as national databases including Scientific Information Database (SID), Indexing Articles Published in Iran Biomedical Journals (IranMedex) and Iranian Magazines Database (Magiran) were used as the source of information. The keywords used for English database search were “Diabetes mellitus” and “Iran*” as the author affiliations. These key terms were used according to each database instructions and were combined using “AND” and “OR” operators appropriately. For search in national databases equivalent Farsi keywords were used. This comprehensive search strategy without any limitation obtained 8668 publications in international journals and 16,921 documents in national journals (a total of 25,589). After assignment, duplications were removed by reviewing the document title as well as the journal volume and issue. As there was a considerable overlap between these databases, the number of obtained documents decreased significantly after removing duplicates. These studies underwent screening based on the abstract and content if required. In this stage, unrelated topics, letter to the editors, meeting abstracts, news, as well as studies on foreign population were excluded and finally 5537 documents remained. In the next step, all obtained articles from databases were merged and categorized according to the articles’ titles to 11 groups as described above. Each category was assigned to a study team member. Two independent researchers performed each step. A third investigator adjudicated discrepancies between the two reviewers.

### Categorization of articles

Then, each working group categorized, assigned documents based on the “Australian Standard Research Classifications”, the “World Health Organization (WHO) classification”, “study design”, and “subject area”.

#### Australian standard research classifications

The Australian research classification system has been in use for several decades and is updated approximately every 10 years to take account of the new and changing field. The National Health and Medical Research Council (NHMRC)’s broad Research Area classification (Basic Science, Clinical Medicine and Science, Health Services Research, Public Health) have been developed by the Australian Bureau of Statistics (ABS) and published as the Australian and New Zealand Standard Research Classification (ANZSRC) [14].

#### WHO classification

Four categories of research are specified in the current typology of health research applicable to WHO [15].

These are:

i) Situation analyses which aims to identify the distribution and determinants of health and disease, and risk factors as well as policy analysis
ii) Health policy and systems research that seeks to improve the delivery, efficiency, effectiveness, equity of health systems, guide health policy development and optimize implementation of health programs;
iii) Product development and intervention research which aims to develop new and improved tools for health promotion and disease control, and to promote implementation research; and
iv) Basic research that aims to advance knowledge of basic biology with particular reference to potential application in tackling human disorders, and to expand human behavior, poverty, dynamics of social organization [14].

#### Study design classification

The study design is the formulation of studies in medical, clinical and other types of research (e.g., epidemiological). There are many ways to classify research designs, but sometimes the distinction is artificial and other times different designs are combined.

Scientific studies can be classified as “Observational Studies”, “Basic Studies”, Experimental (Interventional) Studies”, and “Meta-Analysis – Systematic review” [9].

The list in Table 1 offers a number of useful distinctions between possible research designs:

**Table 1:** Study design classification

Methodology	
Observational studies	Case report/Case series
	Cross sectional
	Case- Control
	Cohort
Basic studies	Immunology
	Pathology
	Biochemistry
	Physiology
Experimental or interventional studies	Randomized controlled trial
	Non-randomized controlled trial
Reviews	Systematic review
	Non- systematic review
Qualitative studies	

Observational studies can be defined as non-interventional and non-experimental [16]. They can be classified as descriptive (Case report, Case series and Cross-sectional) or analytical (Cross-sectional, Case–control, and cohort).

Basic studies focus on in-vitro or in-vivo studies such as animal experiment, genetic and cell studies and also, method development studies. Because the designs of these studies are very different, we’ve put this group of studies in a separate category.

Experimental or interventional studies compare the effect of treatments or interventions with control group. When the preference of participants is not to receive a placebo or control, randomization procedure is not applied. These studies are called Non-Randomized Controlled Studies.

Reviews evaluate evidence of studies conducted in a clinical area. Systematic Review interprets all studies in a determined area. Meta-Analysis uses statistical techniques to synthesize the data from several studies into a single quantitative estimate and provide a clear conclusion.

Qualitative studies are for understanding of underlying reasons, opinions, and motivations. Data collection methods vary by unstructured or semi-structured techniques. Some common methods include focus groups (group discussions), individual interviews, and participation/observations.

#### Subject area classification

According to the research field, each working group categorized documents on top subject areas.

### Trend of publications in the field of diabetes

The number of publications per year was calculated and the proportion of publications within each research area normalized by the total number of publications within the specific research area. This allows the relative growth in each research area to be observed. Research trends in publications in each area were assessed and compared.

### Future activities

The purpose of this project was to establish the priority objectives for diabetes research in Iran, and then to investigate the most realistic and effective ways in which these objectives can be integrated to deliver tangible benefits for people with diabetes. Therefore, to identify potential research gaps and research priorities for each research area, multi- disciplinary expert groups will be invited. The activities of these groups are as follows:

- To determine the similarities and differences between the diabetes research in the world and Iran.
- To recognize the strengths and limitations of diabetes research in Iran.
- Identifying research roadblocks that impede progress across specific areas of diabetes research.
- Assessing ways in which these roadblocks could be overcome.
- Establishing research priorities in each research area for future studies.

Multi- disciplinary expert groups will determine appropriate and realistic goals for patient- centered research in each area of diabetes. They will devise and assess the similarities and differences between the diabetes research in the world and Iran and list the most important scientific advances in individual fields that have an impact on current research. Goals and specific milestones will be considered as a priority and roadblocks that may restrict progress along a given research track will be highlighted and practical strategies to overcome them put in place and new research can be introduced to optimize delivery of the goals. Further, research gaps will be recognized in the research process that can aid planning of further research by reducing duplication of research effort within areas, and focusing research efforts on high priority topics.

After identification of high priority research topics, they will be ranked for future studies of diabetes by multi- disciplinary expert groups. These ranked priorities may be overlap and repetition across and between groups. These groups will also identify comprehensive and useful maps, which will take the appearance of finding gaps and research priorities. These roadmaps are intended to guide future investment in diabetes research in Iran. The roadmaps cover research areas considered by a group of leading experts to offer the greatest scientific interest and potential for advancing the field, ultimately of benefit to the patient.

Research maps developed by each group will be compiled into a single “Iranian Diabetes Research Roadmap” to provide the IDRN to maximize the strengths and opportunities for future diabetes research in Iran. The “Iranian Diabetes Research Roadmap” has produced evidence documents that may be useful to research.

## Conclusion

This project could provide a highly original platform for developing a strategy for diabetes research that is rational and science-based in Iran. The primary goal of the project is to guide choice research areas for future diabetes research. Therefore, for this plan to be successful, it will require monitoring, updating and refining by scientific experts in the diabetes research field so that new and emerging opportunities can be identified. The Iran diabetes roadmap will be updated each 5 years to identify new research gaps.

We will soon provide the highlighted priorities, the gaps of research and strategic mapping of each area of diabetes research that could serve as a precious guideline for national research initiatives in the field of diabetes. By review and assess of strategic mapping, we can achieve many advances in the all aspects of diabetes research that finally impact on the health and well-being of diabetic patients.
